# Reliability of Frozen Section Biopsy in Detecting Endometrial Pathologies: A Systematic Review of Sensitivity and Specificity

**DOI:** 10.7759/cureus.91280

**Published:** 2025-08-30

**Authors:** Mudather Abdelgabar Ali Mohammed, Sumaia Mohammed TalbAllah, Miada Adel Edris Mohamed, Nahla Widatalla Abdalla Hamrawi, Alaa Mohamed Elamin Osman, Tasabeeh Ibrahim Salih Elfaki

**Affiliations:** 1 Anatomical Sciences, St. George’s University, St. George's, GRD; 2 Histopathology, Najran University, Najran, SAU; 3 Acute Medicine, Altnagelvin Area Hospital, Londonderry, IRL; 4 Histopathology, Sudan Medical Specialization Board, Khartoum, SDN; 5 Pathology, Assiut University, Assiut, EGY; 6 Anatomical Sciences, St. George's University School of Medicine, St. George's, GRD

**Keywords:** diagnostic accuracy, endometrial cancer, endometrial hyperplasia, frozen section biopsy, intraoperative diagnosis, sensitivity, specificity

## Abstract

Endometrial pathologies, ranging from benign hyperplasia to carcinoma, present significant diagnostic challenges in gynecologic practice. Frozen section (FS) biopsy, a rapid intraoperative histopathological evaluation method, is widely used for surgical decision-making, but its diagnostic reliability remains debated. This systematic review evaluates the sensitivity, specificity, and overall accuracy of FS biopsy in detecting endometrial pathologies compared with final histopathology.

Following the Preferred Reporting Items for Systematic Reviews and Meta-Analyses (PRISMA) 2020 guidelines, we conducted a comprehensive search of PubMed, Scopus, Web of Science, and Embase up to July 2025. Fifteen studies meeting predefined eligibility criteria (e.g., comparative design, sufficient diagnostic data) were included. Risk of bias was assessed using the Quality Assessment of Diagnostic Accuracy Studies-2 (QUADAS-2) tool. Data on sensitivity, specificity, positive predictive value (PPV), negative predictive value (NPV), and concordance rates were extracted and synthesized narratively due to methodological heterogeneity. Among the 15 studies, FS biopsy exhibited high specificity and variable sensitivity. Concordance with final histopathology was strong for invasive carcinomas but poor for complex atypical hyperplasia (CAH). Reported accuracy ranged from 43.47% to 97%, with higher reliability for high-grade tumors and myometrial invasion. Limitations included sampling errors and pathologist-dependent variability. FS biopsy is highly specific for detecting endometrial malignancies and valuable for intraoperative guidance, particularly when gross lesions are present. However, its limited sensitivity for CAH and early-stage cancers necessitates caution. Integration with preoperative imaging and standardized protocols may optimize its use. Final paraffin-embedded histopathology remains the gold standard for definitive diagnosis.

## Introduction and background

Endometrial pathologies, ranging from benign hyperplasia to endometrial carcinoma, represent a significant burden in gynecologic practice, particularly among perimenopausal and postmenopausal women [1]. Accurate and timely diagnosis of these conditions is crucial for determining appropriate surgical and therapeutic management. In this context, intraoperative decision-making often relies on rapid histological evaluation methods, among which frozen section (FS) biopsy plays a pivotal role [2].

Frozen section biopsy is a diagnostic technique in which surgically removed tissue is quickly frozen, thinly sliced, stained, and examined under a microscope to provide immediate pathological information during surgery [3]. Unlike standard paraffin-embedded histology, which requires more time, FS allows surgeons to receive real-time feedback. Its use is particularly important in gynecologic procedures where an intraoperative diagnosis can determine the surgical approach [4]. For example, if endometrial carcinoma or its high-risk precursors are identified during the operation, the surgeon may proceed with a total hysterectomy and lymphadenectomy rather than opting for a conservative intervention [5].

Despite its widespread use, the diagnostic reliability of FS biopsy in detecting endometrial pathologies remains debated. Studies have reported variable sensitivity (the ability to correctly identify disease) and specificity (the ability to correctly exclude disease), depending on patient population and clinical setting [6,7]. Contributing factors include the heterogeneity of endometrial lesions, tissue artifacts created during freezing, sampling errors, and the experience of the reporting pathologist. Distinguishing between complex atypical endometrial hyperplasia (CAEH) and well-differentiated endometrioid adenocarcinoma remains particularly challenging and can result in misclassification, leading to either overtreatment or underdiagnosis [8].

Given the clinical and surgical implications, it is essential to systematically evaluate the diagnostic performance of FS biopsy in endometrial pathologies. While some meta-analyses and narrative reviews have explored its accuracy, findings remain inconsistent and often limited by variations in methodology. A comprehensive synthesis of recent evidence is therefore warranted to provide greater clarity. This systematic review aims to assess the reliability of frozen section biopsy in diagnosing endometrial abnormalities, focusing on its sensitivity and specificity compared with the final paraffin-embedded histopathological examination, the gold standard. By consolidating current data, this review seeks to refine understanding of FS accuracy, highlight sources of variability, and inform its optimal application in clinical practice.

## Review

Methodology

Study Design and Protocol Registration

This systematic review was conducted in accordance with the Preferred Reporting Items for Systematic Reviews and Meta-Analyses (PRISMA) 2020 guidelines [9]. The review protocol was developed a priori, detailing the objectives, eligibility criteria, data extraction methods, and synthesis strategy. Although the protocol was not registered in PROSPERO, it was strictly adhered to during the conduct of the review to ensure transparency and reproducibility.

Eligibility Criteria

Studies were included in this review based on predefined eligibility criteria framed using the PIRD framework (Population, Index test, Reference standard, Diagnosis). The target population comprised individuals undergoing evaluation for endometrial pathologies. The index test was intraoperative FS biopsy, and the reference standard was final histopathological diagnosis using formalin-fixed paraffin-embedded (FFPE) sections. Only original studies that provided sufficient data to calculate or directly report sensitivity and specificity were considered. Table 1 summarizes the inclusion and exclusion criteria.

**Table 1:** Eligibility Criteria for Inclusion of Studies FS: frozen section.

Inclusion Criteria	Exclusion Criteria
Studies evaluating the diagnostic accuracy of frozen section biopsy for endometrial pathologies	Case reports, reviews, editorials, letters, and conference abstracts
Studies comparing FS biopsy results with final paraffin histopathology	Studies not reporting sensitivity, specificity, or sufficient diagnostic performance data
Human studies published in peer-reviewed journals	Animal studies or in vitro experiments
Articles published in English	Non-English language publications without available English translation
Studies with a clearly defined methodology and sample size	Duplicate studies or overlapping populations (latest or most complete version included)

Information Sources and Literature Search

A comprehensive literature search was conducted across four major electronic databases: PubMed, Scopus, Web of Science, and Embase. The search covered studies published up to July 18, 2025, without any restriction on the starting year of publication. In addition, reference lists of all included studies and relevant review articles were manually screened to identify any additional eligible studies.

The search strategy was developed using a combination of MeSH terms and free-text keywords related to frozen section biopsy and endometrial pathologies. Boolean operators “AND” and “OR” were used to enhance search sensitivity. The detailed search strategy for each database is provided in Table 2.

**Table 2:** Search Strategy

Database	Search Strategy
PubMed	("Frozen Sections"[Mesh] OR "Frozen Section Biopsy" OR "FS biopsy") AND ("Endometrial Neoplasms"[Mesh] OR "Endometrial Carcinoma" OR "Endometrial Hyperplasia" OR "Endometrial Pathology")
Scopus	TITLE-ABS-KEY("frozen section biopsy" OR "intraoperative frozen section") AND TITLE-ABS-KEY("endometrial cancer" OR "endometrial hyperplasia" OR "endometrial pathology")
Web of Science	TS=("frozen section" AND "endometrial") AND TS=("diagnosis" OR "accuracy" OR "sensitivity" OR "specificity")
Embase	('frozen section'/exp OR 'frozen section biopsy') AND ('endometrial cancer'/exp OR 'endometrial hyperplasia' OR 'endometrial disease')

All search results were exported to EndNote X9 reference manager software (Clarivate Analytics, Philadelphia, Pennsylvania). Duplicate entries were identified and removed using the duplicate detection feature. The remaining articles were imported into Microsoft Excel (Microsoft Corporation, Redmond, Washington) for screening and data management.

Selection Process

Two independent reviewers screened the titles and abstracts of all retrieved studies. Full texts of potentially eligible studies were then obtained and assessed against the inclusion criteria. Any disagreements during the screening or full-text review phases were resolved through discussion or consultation with a third reviewer. The study selection process is illustrated using a PRISMA 2020 flow diagram.

Data Collection Process

Data extraction was carried out independently by two reviewers using a standardized and piloted data extraction form. The following information was collected from each included study: author name, year of publication, country, study design, sample size, patient demographics, type of endometrial pathology assessed, FS technique used, reference standard, and diagnostic performance outcomes (true positives, true negatives, false positives, false negatives, sensitivity, specificity).

Discrepancies in data extraction were resolved through discussion, and if necessary, through re-examination of the original full-text articles.

Risk of Bias Assessment

The Quality Assessment of Diagnostic Accuracy Studies 2 (QUADAS-2) tool [10] was used to assess the risk of bias and applicability of the included studies. This tool evaluates studies across four domains: patient selection, index test, reference standard, and flow and timing. Two reviewers independently applied the QUADAS-2 criteria to each study. Disagreements were resolved through consensus. The results of the risk of bias assessment are presented in both tabular and graphical formats.

Synthesis of Results

Given the heterogeneity observed across studies in terms of study design, patient selection, histological classification, frozen section protocols, and reporting standards, a meta-analysis was not conducted. Pooling the sensitivity and specificity data in a quantitative synthesis would have introduced a substantial risk of misleading conclusions due to this clinical and methodological diversity. Moreover, several studies used different criteria for defining positive diagnoses and employed varying levels of pathologist expertise, further limiting the comparability of data.

Instead, a narrative synthesis of the findings was performed, focusing on patterns of diagnostic performance, clinical contexts, and sources of variability across studies.

Results

Study Selection Process

**Figure 1:**
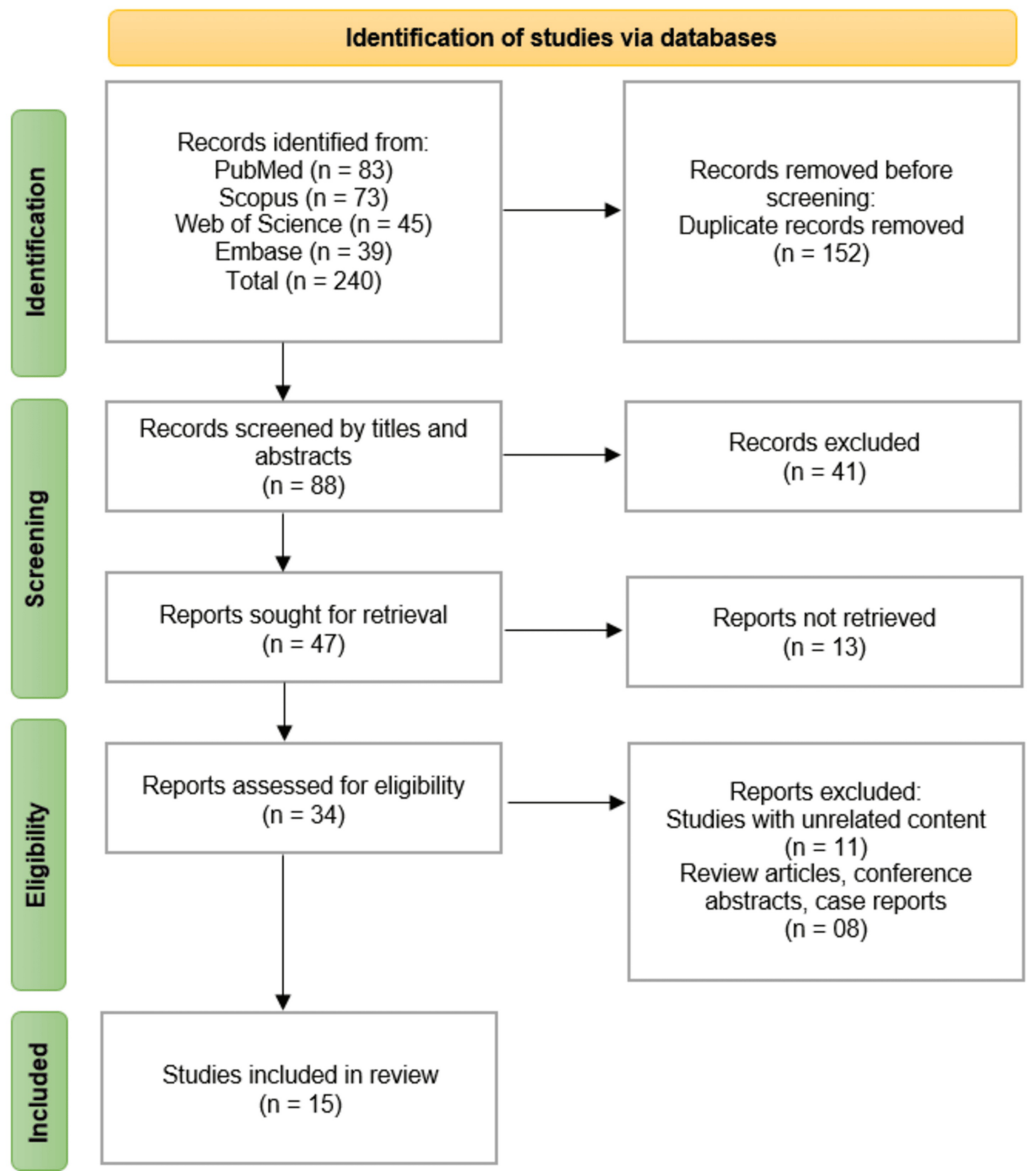
PRISMA Flow Diagram of Studies Selection Process PRISMA: Preferred Reporting Items for Systematic Reviews and Meta-Analyses.

We followed the PRISMA guidelines for study identification and selection. Initially, a comprehensive search across four databases, PubMed (n = 83), Scopus (n = 73), Web of Science (n = 45), and Embase (n = 39), yielded 240 records. After removing 152 duplicate records, 88 studies were screened based on titles and abstracts. Of these, 41 records were excluded for not meeting preliminary criteria. The remaining 47 full-text articles were sought for retrieval, of which 13 could not be accessed. Thus, 34 studies underwent full-text eligibility assessment. After exclusion of 11 studies with unrelated content and 8 review articles, conference abstracts, or case reports, 15 studies [11-25] met the inclusion criteria and were incorporated into the systematic review (Figure 1).

Characteristics of Included Studies

The systematic review included 15 studies [11-25] that evaluated the reliability of FS biopsy in detecting endometrial pathologies, including EIN, endometrial hyperplasia, and EC. These studies were conducted across multiple countries, with the majority from Turkey [11-13,15,18,23,25], followed by the United States [16,19,20,24], and others from India, Spain, Italy, Switzerland, and the Czech Republic [14,17,21,22]. The study designs were predominantly retrospective cohort or observational analyses, with sample sizes ranging from 23 to 868 participants. Mean age was reported in only a few studies, with one noting a mean of 61 years [21]. In most studies, the reference standard was final histopathology, while FS techniques varied and included intraoperative assessments of tumor grade, myometrial invasion, and lymph node status (Table 3).

**Table 3:** Characteristics of Included Studies FS: frozen section, EIN: endometrioid intraepithelial neoplasia, EC: endometrial cancer, IFS: intraoperative frozen section, ACH: atypical complex hyperplasia, CAH: complex atypical hyperplasia, CCC: clear cell carcinoma, CS: carcinosarcoma, UPSC: uterine papillary serous carcinoma, FSA: frozen section analysis, PS: permanent section, FIGO: International Federation of Gynecology and Obstetrics, AEH: atypical endometrial hyperplasia, CAEH: complex atypical endometrial hyperplasia, SEH: simple endometrial hyperplasia, NR: not reported.

Study (Author, Year)	Country	Study Design	Sample Size (n)	Reference Standard	Type of Endometrial Pathologies Assessed	Frozen Section (FS) Technique Used	Main Findings
Ege et al., [11] (2025)	Turkey	Retrospective cohort study	354	Final histopathology of hysterectomy specimens	Endometrioid Intraepithelial Neoplasia (EIN), Endometrial Cancer (EC)	Intraoperative frozen-section assessment	FS accurately identified endometrial carcinoma in EIN cases, particularly in older and postmenopausal women, and effectively guided the need for staging surgery.
Doğan Durdağ et al., [12] (2021)	Turkey	Retrospective observational study	223	Paraffin section	Endometrial cancer – histologic type, grade, tumor diameter, depth of myometrial invasion, cervical and adnexal involvement	Intraoperative FS evaluating histological type, grade, myometrial invasion, tumor diameter, cervical/adnexal spread	FS showed good accuracy but led to some undertreatment. High-risk patients often had lymph node metastasis.
Gokulu et al., [13] (2024)	Turkey	Retrospective cohort study	187	Final pathology report	Endometrioid-type endometrial carcinoma	Intraoperative FS compared with final pathology	Frozen section showed good overall accuracy, with some cases upgraded or downgraded on final pathology.
Limbachiya et al., [14] (2025)	India	Retrospective analysis	100	Final Histopathology	Endometrial carcinoma	IFS	IFS showed high concordance with final histopathology for malignancy, strong agreement for myometrial invasion, accurate grading in high-grade tumors, and reliable lymph node evaluation.
Boyraz et al., [15] (2016)	Turkey	Retrospective cross-sectional study	189	Final pathological examination (post-hysterectomy)	Endometrial hyperplasia ± concurrent endometrial cancer	Intraoperative frozen section during hysterectomy	Frozen section showed high specificity but low sensitivity. Most cancers arose from atypical hyperplasia. Routine use of FS may not be necessary.
Desouki et al., [16] (2017)	USA	Retrospective observational study	250	Final histopathological diagnosis	Endometrial carcinoma (tumor grade, depth of myometrial invasion)	Gross examination, random sections in the absence of gross lesions, and frozen section	FS showed moderate concordance with the final diagnosis for tumor grade. The depth of invasion was often misjudged. Random sections had limited value, while FS was more reliable when a gross lesion was present.
Gallego et al., [17] (2014)	Spain	Prospective	51	Final postoperative pathological evaluation	Endometrial cancer (depth of myometrial invasion)	Intraoperative frozen-section assessment during hysterectomy	FS had 90.2% accuracy, 73.7% sensitivity, 100% specificity; comparable to DWI-MRI with no statistical difference (p = 1)
Gungorduk et al., [18] (2015)	Turkey	Retrospective cohort study	128	Permanent histopathology (paraffin section)	Atypical Complex Hyperplasia (ACH) and Endometrial Carcinoma	Frozen section analysis during total hysterectomy for ACH	Moderate agreement between FS and final diagnosis (κ = 0.61, p < 0.0001); novel preoperative scoring model developed; FS recommended for intraoperative assessment.
Indermaur et al., [19] (2007)	USA	Retrospective Review	23	Final Pathological Diagnosis	Complex Atypical Hyperplasia (CAH), Adenocarcinoma	Intraoperative frozen section	FS correlated with final pathology in 39.1% of cases; 60.8% disagreement; FS is not a reliable indicator for final diagnosis in CAH patients.
Kanis et al., [20] (2016)	USA	Retrospective Analysis	868	Final histology confirmed by a gynecologic pathologist	Grade 3 endometrioid, Clear Cell Carcinoma (CCC), Carcinosarcoma (CS), Uterine Papillary Serous Carcinoma (UPSC)	Frozen Section Analysis (FSA)	FSA improved the detection of high-risk cases missed preoperatively, but was less accurate for certain tumor types.
Kucera et al., [21] (2009)	Prague	Retrospective	63	Permanent Section (PS) Diagnosis	Early-stage Endometrial Cancer (FIGO Stage I)	Intraoperative FS with grading and myometrial invasion assessment	Frozen section biopsy showed high diagnostic reliability with minimal impact on surgical management.
Morotti et al., [22] (2012)	Italy and Switzerland	Retrospective Review	66	Permanent Section Histology	Atypical Endometrial Hyperplasia (AEH) and Endometrial Cancer (EC)	NR	Concordance between FS and permanent section was good (κ=0.75); FS sensitivity 73%, specificity 93.1%, accuracy high; high-risk EC detected more often.
Oz et al., [23] (2014)	Turkey	Retrospective analysis	143	PS	CAEH, SEH, EC	Intraoperative FS during abdominal hysterectomy	FS is reliable for detecting endometrial cancer, but its accuracy depends on the pathologist’s experience.
Stephan et al., [24] (2014)	USA	Retrospective cohort study	116	PS	Endometrioid adenocarcinoma and CAH	Intraoperative frozen section during surgery	High concordance between frozen section and permanent section for histology, grade, and myometrial invasion. Frozen section is reliable for guiding lymphadenectomy decisions.
Turan et al., [25] (2012)	Turkey	Retrospective	125	Paraffin Block Histology	CAEH and Endometrial Cancer	Intraoperative FS examination of hysterectomy samples	FS was Frozen section biopsy showed good agreement with final histology. However, it may not reliably exclude cancer in cases of CAEH.

Diagnostic Performance of Frozen Section Biopsy

The diagnostic performance of FS biopsy was assessed using metrics such as sensitivity, specificity, positive predictive value (PPV), negative predictive value (NPV), and accuracy. Sensitivity ranged widely across studies, from 41.1% [11] to 96.9% [14], while specificity was consistently high, often reaching 100% in studies like Limbachiya et al. [14], Boyraz et al. [15], and Oz et al. [23]. The PPV was also high in several studies, such as 100% in Limbachiya et al. [14] and Gallego et al. [17], indicating a strong ability to correctly identify positive cases. However, NPV varied significantly, with some studies reporting values as low as 12.5% [19], highlighting limitations in ruling out malignancies. Accuracy ranged from 43.47% [19] to 97% [14], with most studies reporting values above 80% (Table 4).

**Table 4:** Diagnostic Performance of Frozen Section Biopsy in Detecting Endometrial Pathologies

Study (Author, Year)	True Positives (TP)	False Positives (FP)	True Negatives (TN)	False Negatives (FN)	Sensitivity (%)	Specificity (%)	PPV (%)	NPV (%)	Accuracy (%)
Ege et al., [11] (2025)	NR	NR	NR	NR	41.1	100	NR	NR	NR
Doğan Durdağ et al., [12] (2021)	NR	NR	NR	4 (undertreated cases)	NR	NR	NR	NR	76.23–95.45 (range across parameters)
Gokulu et al., [13] (2024)	NR	NR	NR	NR	NR	NR	NR	NR	85.6–95.9
Limbachiya et al., [14] (2025)	95	0	5	2	96.9	100	100	40	97
Boyraz et al., [15] (2016)	6	1	35	5	54.5	97.2	85.7	87.5	89.1
Desouki et al., [16] (2017)	NR	NR	NR	NR	NR	100% (gross-only cases)	NR	85% (gross-only cases)	NR
Gallego et al., [17] (2014)	14	0	32	5	73.7	100	100	86.5	90.2
Gungorduk et al., [18] (2015)	NR	NR	NR	NR	80.9	70.3	75.3	76.4	NR
Indermaur et al., [19] (2007)	9	6	1	7	56.25	14.29	60.00	12.50	43.47
Kanis et al., [20] (2016)	NR	NR	NR	NR	77	95	NR	NR	NR
Kucera et al., [21] (2009)	NR	NR	NR	NR	77	95	NR	NR	NR
Morotti et al., [22] (2012)	27	2	27	10	73	93.1	93.1	73	81.8
Oz et al., [23] (2014)	43	0	83	17	71.6% (≈76%)	100%	100%	83	88
Stephan et al., [24] (2014)	NR	NR	NR	7	NR	NR	NR	NR	NR
Turan et al., [25] (2012)	60	1	47	14	81.1	97.9	98.4	76.7	85.6

Concordance Between Frozen Section and Final Histopathology

The concordance between FS and final histopathology was evaluated in several studies. For instance, Limbachiya et al. [14] reported a 97% concordance rate for malignancy detection, while Morotti et al. [22] noted good agreement (κ=0.75) between FS and permanent sections. However, discrepancies were observed in specific scenarios, such as CAH, where FS demonstrated poor reliability [19]. Studies such as Gungorduk et al. [18] and Oz et al. [23] emphasized that FS accuracy depended largely on the pathologist’s experience and the presence of gross lesions.

Limitations and Variability in Findings

The reliability of FS biopsy varied depending on the endometrial pathology assessed. For EC, FS demonstrated high accuracy in detecting high-grade tumors and myometrial invasion [14,24]. However, its performance was less reliable for CAH and early-stage cancers, with some studies reporting undertreatment or misclassification [12,19]. Additionally, FS proved more reliable when gross lesions were present, whereas random sections of normal-appearing endometrium had limited diagnostic value [16].

Results of Risk of Bias Assessment

Most studies demonstrated a low risk of bias across all domains [11,13,14,17,20-23], as they employed clear selection criteria, standardized FS techniques, and reliable final histopathology confirmation. However, a moderate risk of bias was observed in Doğan Durdağ et al. [12] and Desouki et al. [16] due to unclear patient selection criteria, as well as in Boyraz et al. [15] and Gungorduk et al. [18] because of their focus on specific subgroups (hyperplasia and atypical complex hyperplasia, respectively). Only one study, Indermaur et al. [19], exhibited a high risk of bias due to small sample size, high false-negative rates, and poor concordance between FS and final pathology in CAH cases. Overall, the majority of studies (9/15) were methodologically robust, supporting the reliability of their diagnostic accuracy findings, while the remaining studies warrant cautious interpretation due to potential biases (Table 5).

**Table 5:** Risk of Bias Assessment (QUADAS-2)

Study (Author, Year)	Patient Selection	Index Test (FS Biopsy)	Reference Standard	Flow & Timing	Overall Risk of Bias
Ege et al., [11] (2025)	Low	Low	Low	Low	Low
Doğan Durdağ et al., [12] (2021)	Unclear	Low	Low	Low	Moderate
Gokulu et al., [13] (2024)	Low	Low	Low	Low	Low
Limbachiya et al., [14] (2025)	Low	Low	Low	Low	Low
Boyraz et al., [15] (2016)	High	Low	Low	Low	Moderate
Desouki et al., [16] (2017)	Unclear	Low	Low	Low	Moderate
Gallego et al., [17] (2014)	Low	Low	Low	Low	Low
Gungorduk et al., [18] (2015)	Moderate	Low	Low	Low	Moderate
Indermaur et al., [19] (2007)	High	High	Low	High	High
Kanis et al., [20] (2016)	Low	Low	Low	Low	Low
Kucera et al., [21] (2009)	Low	Low	Low	Low	Low
Morotti et al., [22] (2012)	Low	Low	Low	Low	Low
Oz et al., [23] (2014)	Low	Low	Low	Low	Low
Stephan et al., [24] (2014)	Low	Low	Low	Low	Low
Turan et al., [25] (2012)	Low	Low	Low	Low	Low

Discussion

This review comprehensively evaluated the reliability of FS biopsy in detecting endometrial pathologies, incorporating 15 studies that collectively examined its diagnostic performance across various clinical contexts. The findings reveal both the strengths and limitations of FS biopsy, offering critical insights into its role in intraoperative decision-making for EC, EIN, and atypical hyperplasia. The results demonstrate that FS biopsy exhibits high specificity and variable sensitivity, with its accuracy heavily influenced by the type of endometrial pathology, the presence of gross lesions, and the experience of the pathologist. These observations align with prior literature but also highlight significant discrepancies that warrant careful consideration in clinical practice.

One of the most consistent findings across the included studies was the high specificity of FS biopsy, particularly for detecting endometrial malignancies. Studies such as Limbachiya et al. [14], Boyraz et al. [15], and Oz et al. [23] reported specificity values of 100%, indicating that FS biopsy rarely misclassifies benign lesions as malignant. This is clinically significant, as high specificity reduces the risk of unnecessary surgical interventions, such as lymphadenectomy, in patients without aggressive disease. The high PPV observed in these studies further supports the utility of FS biopsy for confirming malignancy when gross lesions are present. For instance, Gallego et al. [17] noted a PPV of 100%, suggesting that a positive FS result reliably predicts the presence of EC. These findings are consistent with earlier research by Kopatsaris et al. [26], who similarly reported that FS biopsy excels in ruling in malignancy but may struggle with ruling out benign or premalignant conditions.

However, the sensitivity of FS biopsy exhibited considerable variability, ranging from 41.1% [11] to 96.9% [14]. This wide range underscores the technique's limitations in detecting certain endometrial pathologies, particularly early-stage cancers and CAH. For example, Indermaur et al. [19] reported a sensitivity of only 56.25% for CAH, with a high false-negative rate (60.8% discordance with final histopathology). This aligns with the work of Kamoi et al. [27], who argued that FS biopsy is less reliable for diagnosing premalignant lesions due to sampling errors and the subjective interpretation of nuclear atypia. The low NPV in some studies, such as 12.5% in Indermaur et al. [19], further emphasizes that FS biopsy cannot reliably exclude malignancy in cases of CAH or early EC. This has important implications for clinical practice, as it suggests that FS biopsy may not be sufficient as a standalone diagnostic tool for these conditions, and final histopathology should remain the gold standard.

The concordance between FS and final histopathology was another critical theme in this review. Studies like Limbachiya et al. [14] and Morotti et al. [22] reported high concordance rates (97% and κ=0.75, respectively) for detecting malignancy and assessing myometrial invasion. These results are comparable to those of a meta-analysis by Pecorelli et al. [28], which found that FS biopsy had an overall accuracy of 89% for EC staging. However, the review also identified scenarios where FS biopsy underperformed. For instance, Desouki et al. [16] noted that FS biopsy often misjudged the depth of myometrial invasion, particularly in cases where gross lesions were absent. This echoes the findings of Tanaka et al. [29], who reported that FS biopsy was less accurate than MRI for assessing deep myometrial invasion, a critical factor in surgical planning. These discrepancies suggest that while FS biopsy is valuable for initial intraoperative assessment, it should be complemented with imaging or final pathology for definitive staging.

The role of pathologist expertise and the presence of gross lesions emerged as key factors influencing FS accuracy. Oz et al. [23] and Gungorduk et al. [18] both emphasized that FS reliability improved when performed by experienced pathologists and when gross lesions were visible. This is consistent with the broader literature on diagnostic variability in surgical pathology, where interobserver agreement is known to vary significantly based on training and case volume [30]. For example, Gungorduk et al. [18] developed a preoperative scoring model to improve FS accuracy in atypical hyperplasia cases, highlighting the potential for standardized protocols to mitigate diagnostic inconsistencies. However, reliance on gross lesions also introduces a limitation, as Desouki et al. [16] found that random sections of normal-appearing endometrium had limited diagnostic value. This raises questions about the generalizability of FS biopsy in cases where macroscopic abnormalities are absent, a challenge also noted by Trimble et al. [31] in their study of endometrial sampling techniques.

The risk of bias assessment revealed that most studies (9/15) were methodologically robust, with low risk of bias across all QUADAS-2 domains. These studies, including Ege et al. [11] and Stephan et al. [24], employed clear selection criteria and standardized FS techniques, enhancing the reliability of their findings. However, a moderate risk of bias was identified in studies like Doğan Durdağ et al. [12] and Boyraz et al. [15], primarily due to unclear patient selection or focus on specific subgroups. The only study with a high risk of bias, Indermaur et al. [19], was limited by its small sample size and high false-negative rate, which may have skewed its results. These methodological variations underscore the need for caution when interpreting findings across studies, particularly those with potential biases.

When compared with existing literature, the results of this review both corroborate and challenge prior conclusions. For instance, the high specificity and PPV of FS biopsy align with earlier systematic reviews by Proctor et al. [32], who advocated for its use in intraoperative EC staging. However, the variable sensitivity and NPV observed in this review contrast with some optimistic claims in the literature, such as those by Zhong et al. [33], who suggested that FS biopsy could replace final pathology in certain cases. Instead, the present findings support a more nuanced view, where FS biopsy serves as a valuable adjunct but not a replacement for comprehensive histopathological evaluation. This is particularly relevant for high-risk populations, where studies like Kanis et al. [20] and Stephan et al. [24] demonstrated that FS biopsy could identify aggressive tumor types but might miss subtle features of early disease.

The clinical implications of these findings are multifaceted. On one hand, FS biopsy offers real-time diagnostic information that can guide surgical decisions, such as the extent of lymphadenectomy or the need for staging procedures. This is especially useful in resource-limited settings where rapid turnaround times are critical. On the other hand, its limitations in diagnosing CAH and early EC suggest that clinicians should exercise caution when relying solely on FS results for these conditions. Integrating FS biopsy with preoperative imaging, such as MRI or ultrasound, could mitigate some of these limitations, as proposed by Gallego et al. [17]. Additionally, the development of standardized protocols and training programs for pathologists, as suggested by Gungorduk et al. [18], could further enhance the reliability of FS biopsy.

Limitations

Despite its comprehensive scope, this review has several limitations. First, the predominance of retrospective studies introduces potential biases, such as selection bias and incomplete data reporting. Second, the heterogeneity in study designs, sample sizes, and FS techniques limits the ability to perform a meta-analysis or draw uniform conclusions. Third, the focus on diagnostic accuracy metrics without detailed clinical outcome data (e.g., long-term survival or recurrence rates) restricts the assessment of the FS biopsy's broader impact on patient care. Finally, the exclusion of non-English studies may have omitted relevant data from other regions, potentially affecting the generalizability of the findings.

## Conclusions

This study highlights the dual nature of frozen section biopsy as both a valuable intraoperative tool and a technique with notable limitations. Its high specificity and PPV make it reliable for confirming malignancy, particularly in the presence of gross lesions, but its variable sensitivity and NPV caution against overreliance in diagnosing premalignant or early-stage conditions. The concordance with final histopathology is generally strong for aggressive tumors but weaker for subtle or heterogeneous lesions. Moving forward, efforts to standardize FS protocols, improve pathologist training, and integrate multimodal diagnostic approaches could enhance its accuracy and clinical utility. Until then, FS biopsy should be viewed as a complementary-rather than definitive-diagnostic method, with final histopathology remaining the gold standard for endometrial pathology assessment.
